# Minimal-invasive periazetabuläre Osteotomie zur Therapie der Hüftdysplasie des Erwachsenen

**DOI:** 10.1007/s00064-022-00771-w

**Published:** 2022-05-18

**Authors:** Georgi I. Wassilew, Andre Hofer, Anastasia Rakow, Sebastian Gebhardt, Manuela Hoffmann, Viktor Janz, Alexander Zimmerer

**Affiliations:** 1 Klinik und Poliklinik für Orthopädie und Orthopädische Chirurgie, Universitätsmedizin Greifswald, 17475 Greifswald, Deutschland; 2 ARCUS Kliniken, Rastatterstr. 17–19, 75179 Pforzheim, Deutschland

**Keywords:** Beckenosteotomie, Minimal-invasiv, M. sartorius erhaltend, Residuelle Hüftdysplasie, Hüftgelenkerhaltende Chirurgie, Pelvic osteotomy, Residual/developmental dysplasia of the hip, Sartorius muscle sparing, Hip preservation surgery, Minimally invasive surgery

## Abstract

**Operationsziel:**

Durchführung einer periazetabulären Osteotomie (PAO) über einen minimal-invasiven Zugang zur dreidimensionalen Korrektur der Orientierung der Hüftgelenkpfanne.

**Indikationen:**

Symptomatische Hüftdysplasie in der Adoleszenz nach dem Schluss der Y‑Wachstumsfuge und beim Erwachsenen.

**Kontraindikationen:**

Fortgeschrittene Arthrose (Arthrosegrad ≥ 2 nach Tönnis), präformierte Sekundärpfanne, offene Y‑Wachstumsfuge.

**Operationstechnik:**

Über einen minimal-invasiven Zugang wird eine periazetabuläre Osteotomie durchgeführt.

**Ergebnisse:**

Insgesamt wurden 39 Patienten über 3,5 (3 bis 4,5) Jahre nachbeobachtet. Der laterale Centrum-Erker-Winkel nach Wiberg konnte signifikant von 16,1° (7–24°) auf 30,5° (25–37°) (*p* < 0,0001), der Tragflächenwinkel von 13,2° (2–25,3°) auf 2,8° (−3–13°) (*p* < 0,0001) korrigiert werden. Die mittlere Operationszeit betrug 88 (57 bis 142) Minuten. Es traten keine schweren Komplikationen auf.

## Abstract (en)

**Objective:**

Performance of a periacetabular osteotomy (PAO) using a minimally invasive approach for three-dimensional correction of the acetabular position.

**Indications:**

Symptomatic developmental dysplasia of the hip in adolescents and adults.

**Contraindications:**

Advanced osteoarthritis of the hip, incongruence of the hip joint surfaces.

**Surgical technique:**

A periacetabular osteotomy is performed via a minimally invasive approach.

**Results:**

In total, 39 patients were followed up for 3.5 (3–4.5) years. The lateral center-edge angle of Wiberg increased significantly from 16.1° (7–24°) to 30.5° (25–37°) (*p* < 0.0001), the acetabular index changed from 13.2° (2–25.3°) to 2.8° (−3–13°; *p* < 0.0001). Mean duration of surgery was 88 (57–142) minutes. No major complications occurred.

## Vorbemerkungen

Die Hüftdysplasie ist durch eine unzureichende azetabuläre Überdachung des Femurkopfes gekennzeichnet, die zu einer pathologischen Druckerhöhung und einer Überlastung des lateralen Knorpel-Labrum-Komplexes führt. Dies kann eine Degeneration des Hüftgelenks und eine vorzeitige sekundäre Arthrose bedingen [4, 7, 11].

Um die adulte Hüftdysplasie zu behandeln, wurden in den vergangenen Dekaden verschiedene Techniken der Beckenosteotomien beschrieben, wie etwa die Dreifachbeckenosteotomie nach Tönnis, die Rotationsosteotomie nach Ninomiya und die Birmingham Interlocking-Osteotomie [8, 12, 16]. Neben vielen Vorteilen sind diese Techniken jedoch auch mit verschiedenen Einschränkungen und Nachteilen assoziiert. So engt z. B. die Dreifachbeckenosteotomie nach Tönnis den Geburtskanal ein und ist mit einer langen Rekonvaleszenz assoziiert [10, 14, 19]. Die Rotationsosteotomie nach Ninomiya ist vergleichsweise invasiv, technisch sehr anspruchsvoll und ebenfalls mit einer langen Rekonvaleszenz verbunden [12].

Die Arbeitsgruppe um Reinhold Ganz beschrieb 1988 die periazetabuläre Osteotomie (PAO) zur dreidimensionalen Korrektur des Azetabulums [3]. Zu den Vorteilen dieser Technik gehören das gute dreidimensionale Korrekturpotenzial bei hoher postoperativer Stabilität des Beckens durch Erhaltung der Kontinuität des hinteren Hüftpfannenpfeilers, welche eine schnelle Mobilisierung nach der Operation erlaubt [2, 10, 15, 19]. Außerdem wird der innere Beckendurchmesser erhalten, was eine natürliche Geburt ermöglicht [2, 10, 15, 19]. Im Vergleich mit anderen Beckenosteotomien wird für die PAO eine geringere Pseudarthroserate postuliert, die auf die erhöhte postoperative Beckenstabilität und die größeren spongiösen Kontaktflächen zurückgeführt werden könnte [19]. Diese Faktoren könnten neben der raschen Verheilung der Osteotomien eine schnelle Rekonvaleszenz nach PAO begünstigen.

Die PAO ist ein chirurgischer Eingriff, der bei zumeist jungen Erwachsenen mit symptomatischer Hüftdysplasie mit dem primären Ziel der Schmerzreduktion durchgeführt wird. Es wurden endoprothesenfreie „Überlebensraten“ von ca. 88 % nach 10 Jahren, 61 % nach 20 Jahren und 29 % nach 30 Jahren berichtet [9]. Diese Ergebnisse basieren jedoch auf einer relativ kleinen Kohorte von 63 Patienten (75 Hüften) und den initialen Erfahrungen mit dieser Technik [9]. Weitere 20- bis 30-Jahres-Überlebensraten anderer Zentren mit größeren Kohorten, die mit einer weiterentwickelten Technik der PAO behandelt wurden, stehen jedoch noch aus.

Die ursprüngliche chirurgische Technik der PAO war mit einer ausgedehnten Hautinzision und mit der Ablösung verschiedener Muskelursprünge (vollständig des M. sartorius, des M. rectus femoris und anteilig des M. obliquus internus abdominis, des M. transversus abdominis, M. tensor fasciae latae, der Mm. glutei medius und minimus) assoziiert [3]. Diese Technik wurde über die letzten Dekaden von der erstbeschreibenden Berner Arbeitsgruppe modifiziert [1]. So ermöglichen minimal-invasive Techniken (MIS-PAO) kürzere Hautschnitte und/oder günstigere Inzisionsverläufe, eine reduzierte Ablösung oder den Erhalt der Muskelansätze [13] und somit die Minimierung des Weichteiltraumas, was potenziell zu einer schnelleren postoperativen Genesung des Patienten beitragen könnte [2, 6, 17, 18].

Verglichen mit der konventionellen PAO und den bisher publizierten minimal-invasiven Techniken zeigt die hier beschriebene Technik einige Unterschiede. So wird in der hier beschriebenen Technik ein Bikini-Hautschnitt verwendet und der M. sartorius wird weder abgelöst noch gespalten. Bei der konventionellen PAO wird traditionell der modifizierte Smith-Petersen-Zugang verwendet [7]. Bestandteil der von uns modifizierten, minimal-invasiven PAO-Technik ist ein Bikini-Hautschnitt, ähnlich der Schnittführung des ilioinguinalen Zugangs, welcher parallel zu den Hautspannungslinien verläuft und daher zu günstigeren kosmetischen Ergebnissen führt. Die aktuellen in der Literatur beschriebenen MIS-PAOs verwenden eine den M. rectus femoris erhaltende Technik, wobei der Ursprung dieses Muskels an der Spina iliaca anterior inferior geschont wird [2, 18].

Während bei der Mehrzahl dieser MIS-PAOS der M. sartorius an seinem Ursprung abgelöst wird, beschrieb eine dänische Arbeitsgruppe die Spaltung des M. sartorius (transsartorialer Zugang) medial von der Spina iliaca anterior superior und im Faserverlauf nach distal für den Zugang zu den Sitzbein- und Schambeinosteotomien [17]. Der N. femoralis cutaneus lateralis (NCFL) tritt kurz vor der Spina iliaca anterior superior durch die Fascia iliaca und gleich danach unterhalb des Leistenbandes über den M. sartorius an den lateralen Oberschenkel. Damit kreuzt der transsartoriale Zugang den Verlauf des NCFL, was mit einem hohen Verletzungsrisiko assoziiert ist.

In der hier vorgestellten MIS-PAO-Technik werden sowohl der Ursprung des M. rectus femoris als auch der Ursprung des M. sartorius geschont. Der M. sartorius wird nicht gespalten, sondern in seiner muskulären Integrität erhalten und für den Zugang zu den Osteotomieebenen am Sitzbein und Schambein nach medial mobilisiert.

Somit ist nach gegenwärtigem Kenntnisstand die hier beschriebene MIS-PAO eine der am wenigsten invasiven Techniken. Diese Technik wird nun im Folgenden im Detail dargestellt.

## Operationsprinzip und -ziel

Minimal-invasive Durchführung einer PAO unter maximal muskelschonender Präparation, ohne Ablösung der Ansätze der Mm. sartorius und rectus femoris.

## Vorteile

Potenziell bestehen im Vergleich zur konventionellen PAO folgende Vorteile:

- kosmetisch günstigere Ergebnisse (schmale Narbe),
- geringeres Weichteiltrauma,
- schnellere Rekonvaleszenz/Rehabilitation nach der Operation.

## Nachteile

Potenziell bestehen im Vergleich zur konventionellen PAO folgende Nachteile:

- Gefahr der Verletzung des N. cutaneus femoris lateralis (NCFL), die zu einer entsprechenden temporären oder permanenten Dys‑/Hypoästhesie im Versorgungsgebiet führen kann,
- schlechtere/diffizilere direkte Visualisierung des Operationssitus.

## Indikationen

- Radiologische Diagnose einer Hüftdysplasie (LCE-Winkel < 25°, Tragflächenwinkel ≥ 14°, Femurkopfextrusionsindex > 27 %)
- Klinisch symptomatische Hüftdysplasie mit:
  - belastungsabhängigem Leistenschmerz,
  - Schmerz im Bereich des Trochanter major (aufgrund einer Überlastung der Abduktoren),
  - meist erhöhtem Bewegungsumfang (v. a. eine erhöhte Innenrotation bei 90° Hüftflexion),
  - mechanischen Symptomen wie „Klicken“ und „Schnappen“,
  - Hinken (selten).

## Kontraindikationen

- Osteoarthrose im fortgeschrittenen Stadium (Arthrosegrad ≥ 2 nach Tönnis)
- Offene Y‑Wachstumsfuge
- Subluxation des Hüftkopfes mit Bildung einer Sekundärpfanne
- Nicht korrigierbare Inkongruenz zwischen Hüftkopf und Azetabulum (radiologische Funktionsaufnahmen werden hier zwingend empfohlen)
- Ausgeprägte Osteoporose, etwa bei Patienten mit Zerebralparese

## Patientenaufklärung

- Verletzung des N. cutaneus femoris lateralis
- Gefäß- und Nervenverletzungen (Aa./Vv./Nn. femoralis, obturatorius, ischadicus)
- Über- oder Unterkorrektur
- Wundheilungsstörung und Infektionen
- Beschwerdepersistenz
- Postoperative Adhäsionen
- Postoperative heterotope Ossifikationen
- Pseudarthrose des Os pubis bei größeren Korrekturen, die meist asymptomatisch sind
- Pseudarthrose des Os ischium und Ermüdungsfraktur des unteren Schambeinastes bei intraoperativer Perforation oder postoperativer Fraktur der hinteren Säule

## Operationsvorbereitungen

Präoperativ sind eine gründliche Anamnese und eine körperliche Untersuchung obligat. Die körperliche Untersuchung sollte eine sorgfältige Beurteilung des Gangbildes, der Beinlängen, der Gelenkstabilität und des Bewegungsumfangs umfassen.

Es sollten korrekte Röntgenaufnahmen angefertigt werden, um den Grad der Hüftdysplasie exakt beurteilen und die Korrektur planen zu können. Es sollten eine anterior-posteriore (a.-p.) Beckenübersicht (in korrekter Rotation und Neigung) und eine Dunn-Aufnahme der betroffenen Hüfte durchgeführt werden. Ergänzend können Funktionsaufnahmen z. B. in Abduktion und Innenrotation angefertigt werden.

**Figure 1:**
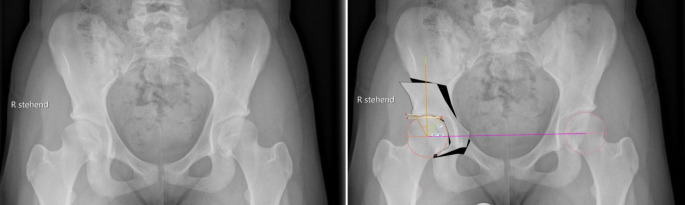

Vor der Operation ist die digitale Planung obligat, um das optimale Ausmaß der Korrektur zu bestimmen und eine postoperative Über- oder Unterkorrektur zu vermeiden. In der a.-p.-Beckenübersicht sind ein postoperativer LCE-Winkel von 30°, ein positiver Tragflächenwinkel (Norm zwischen 3 und 13°), ein Femurkopfextrusionsindex ≤ 27 % (Norm von 17° und 27°) und ein antevertiertes Azetabulum die Zielwerte für die Korrektur (Abb. 1). Die azetabuläre Version kann aktuell jedoch nicht mittels der Planungssoftware geplant werden. Diese sollte jedoch präoperativ evaluiert werden, und in diesem Zusammenhang sollten präoperativ der „acetabular wall index“ und Retroversionszeichen wie „cross-over sign“, „posterior wall sign“ und der „acetabular wall index“ erhoben werden.

Außerdem sollte, wenn möglich, eine mediale Dysplasie vermieden werden. Bei sehr kurzer azetabulärer Knorpeltragfläche muss individuell ein Kompromiss zwischen medialem und lateralem CE-Winkel sowie dem Tragflächenwinkel gefunden werden, um eine nahezu „normale“ Überdachung zu erreichen.

Außerdem sollten eine hochauflösende radiäre Magnetresonanztomographie (MRT) für die Diagnostik intraartikulärer Schäden und Asphärizitäten des Kopf-Schenkelhals-Übergangs sowie eine Rotations-MRT (Hüftgelenk-Kniegelenk) für die Erfassung der femoralen Antetorsion durchgeführt werden. Während eine Formstörung des Kopf-Schenkelhals-Übergangs intraoperativ diagnostiziert werden kann, muss eine Retrotorsion des Schenkelhalses zwingend präoperativ diagnostiziert werden, um ein postoperatives sekundäres femoroazetabuläres Impingement und eine schlechte Funktion zu vermeiden.

## Instrumentarium

**Figure 2:**
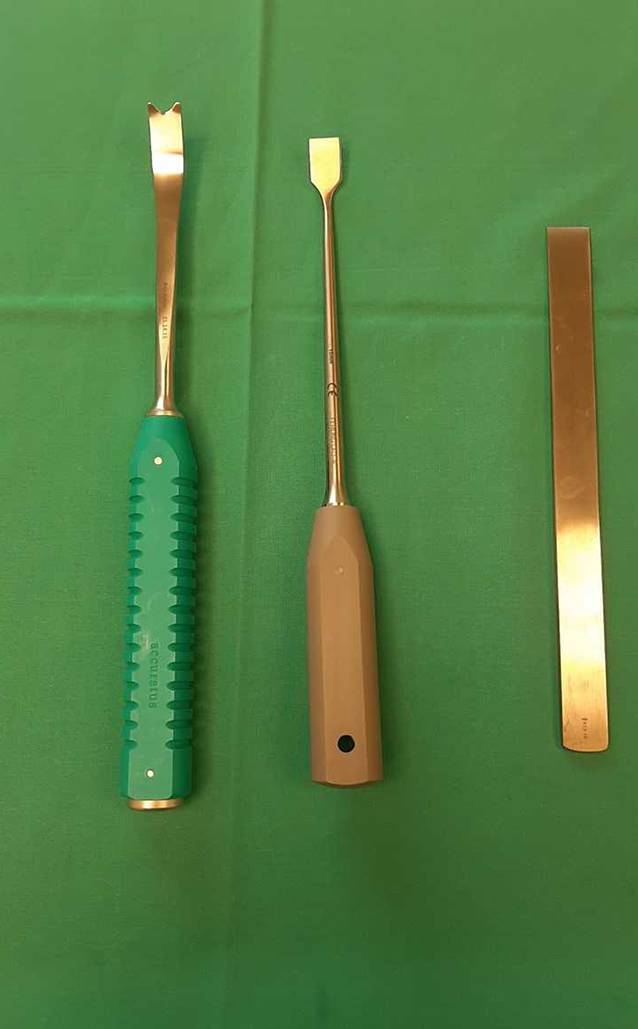

- Pinzetten und Skalpell
- Cobb-Raspatorium
- 2 spitze und 3 stumpfe Hohmann-Hebel
- 2 Roux-Haken
- Spezielle gerade und gebogene Osteotome (nach Ganz, nach Lambotte; Abb. 2)
- Oszillierende Säge
- 2 Schanz-Schrauben mit T‑Handgriff
- Laminaspreizer
- 4,5-mm-Kortikalisschrauben
- Gegebenenfalls Hochgeschwindigkeitsfräse zur Durchführung einer Schenkelhalsmodulation

## Anästhesie und Lagerung

- Allgemeinanästhesie, ggf. ergänzend regionale Verfahren (z. B. Transversus-abdominis-plane-Block, PDK), ggf. PDA-Pumpe zur postoperativen Analgesie
- Rückenlagerung, wobei der kontralaterale Arm auf einer Armstütze ausgelagert und der ipsilaterale Arm an einem Armhalter über der Kopf‑/Halsregion hängend gelagert wird

## Operationstechnik

(Abb. 3, 4, 5, 6, 7, 8, 9, 10, 11, 12, 13, 14, 15, 16 und 17).

**Figure 3:**
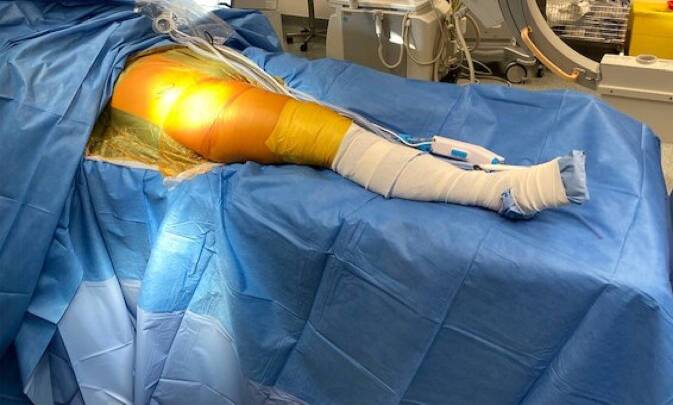

**Figure 4:**
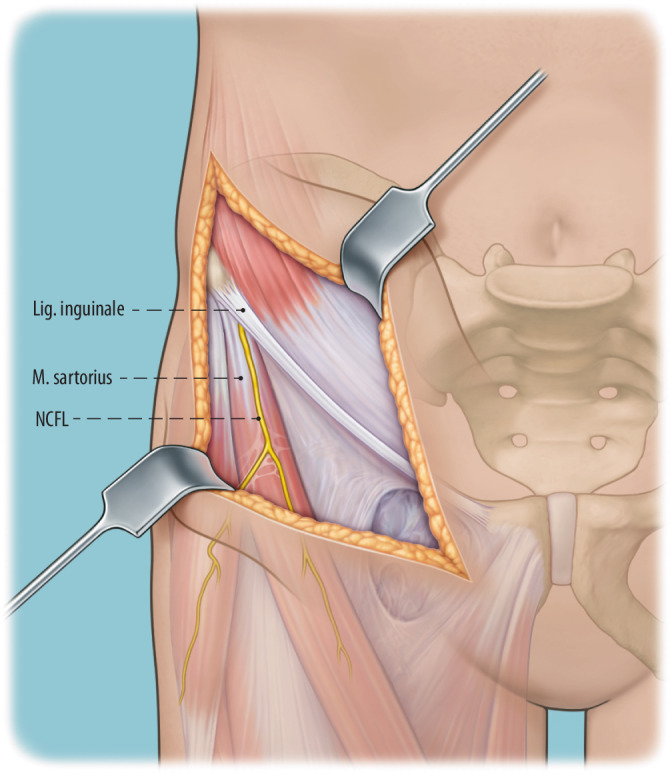

**Figure 5:**
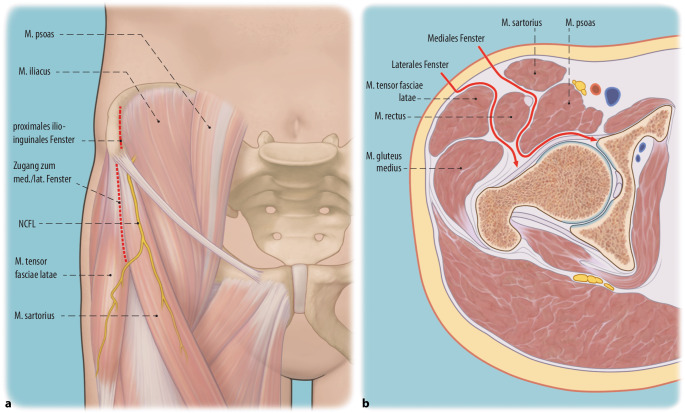

**Figure 6:**
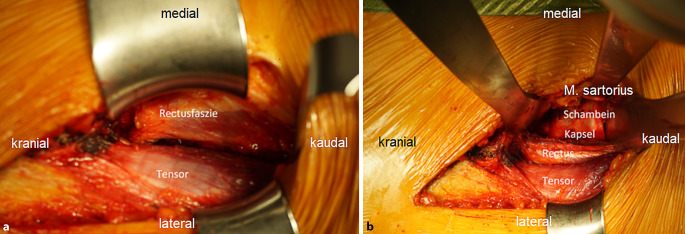

**Figure 7:**
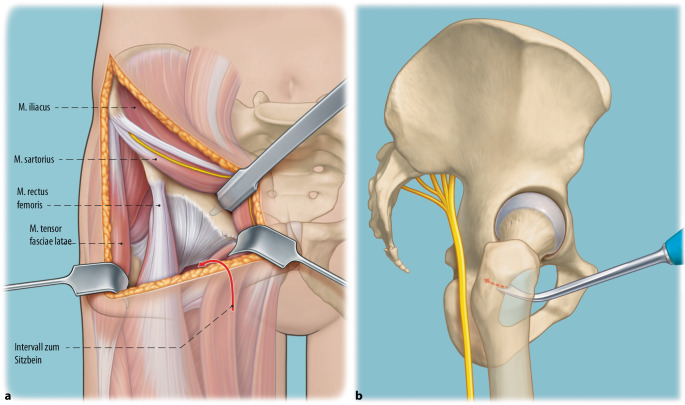

**Figure 8:**
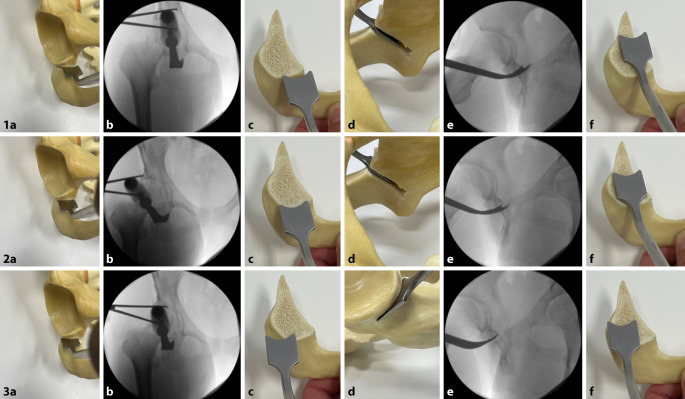

**Figure 9:**
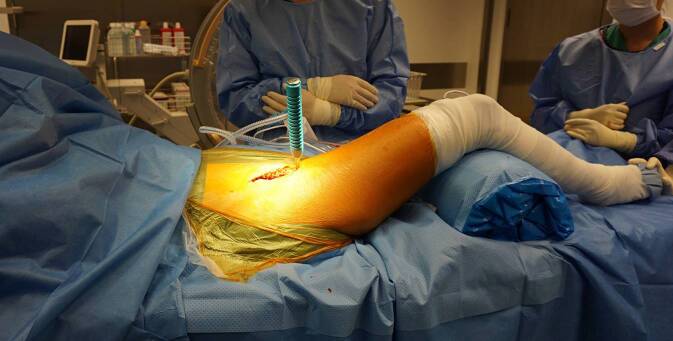

**Figure 10:**
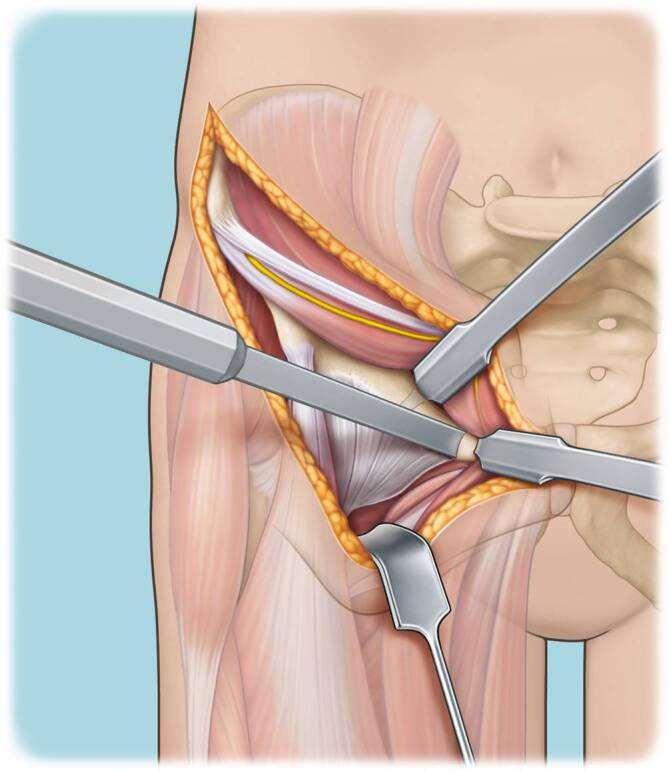

**Figure 11:**
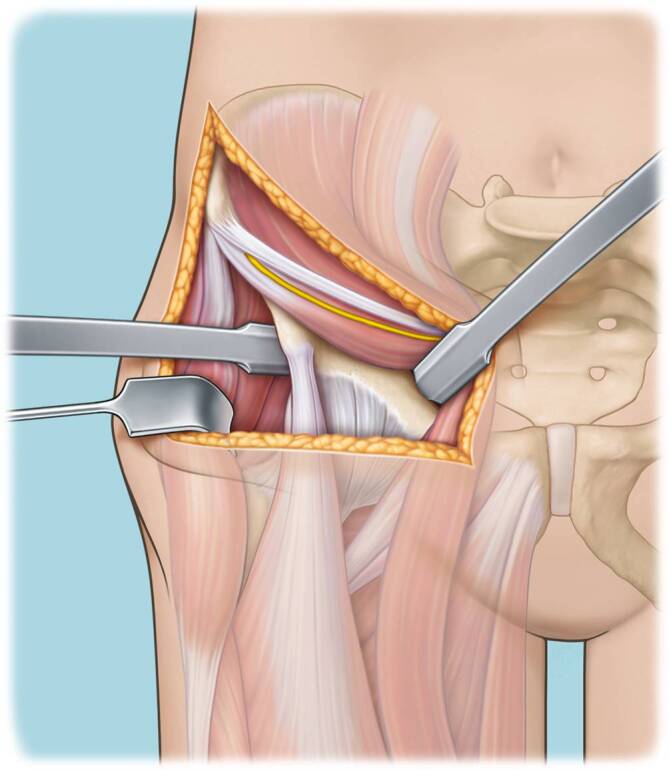

**Figure 12:**
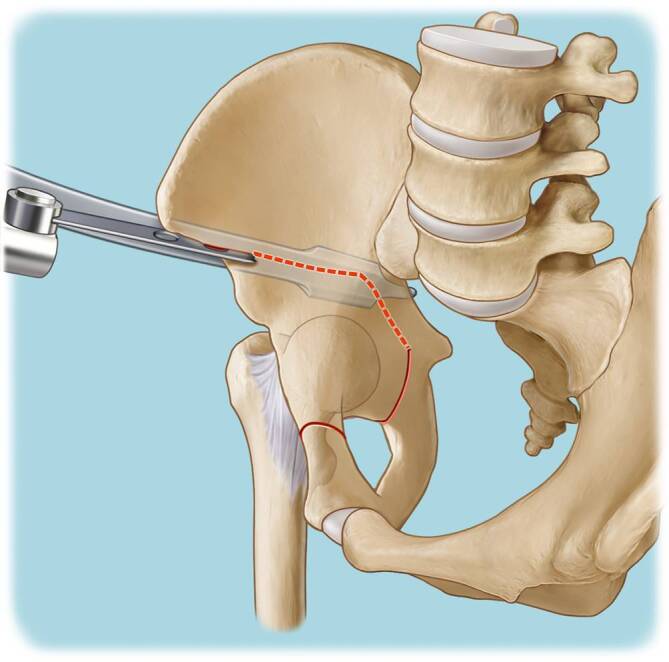

**Figure 13:**
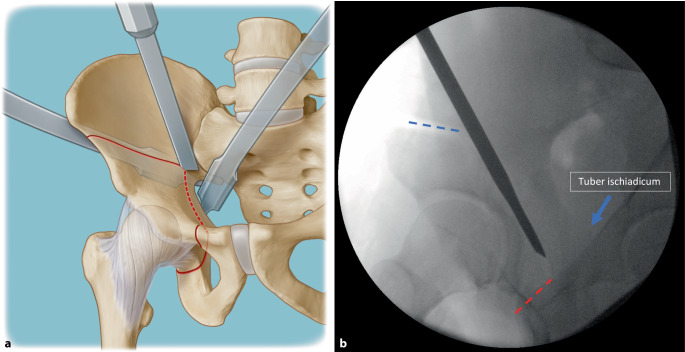

**Figure 14:**
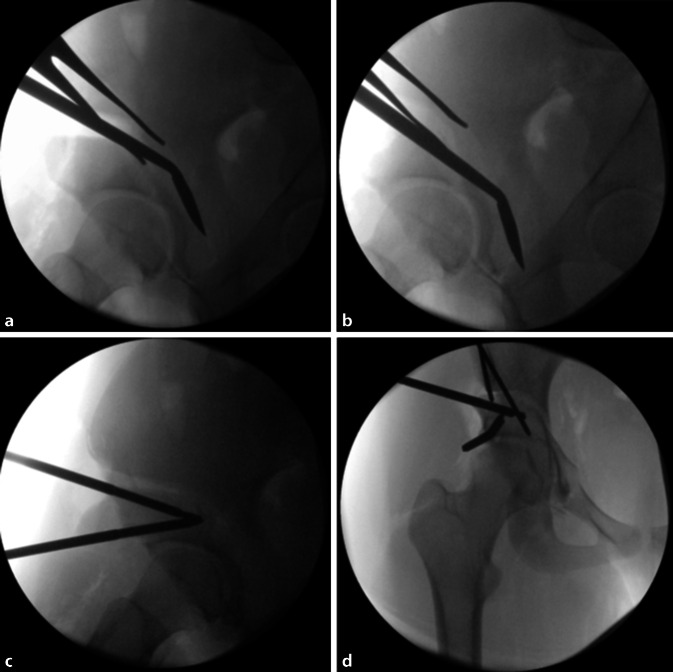

**Figure 15:**
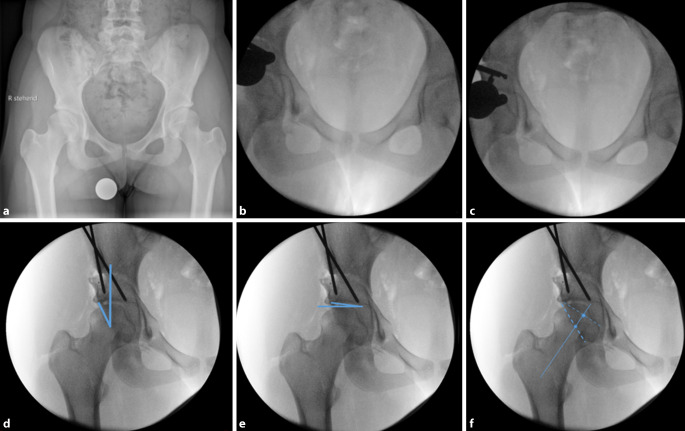

**Figure 16:**
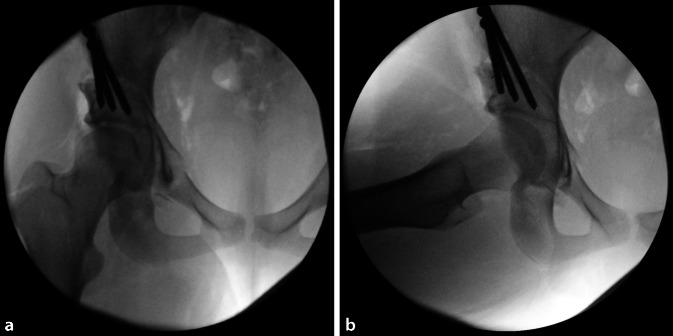

**Figure 17:**
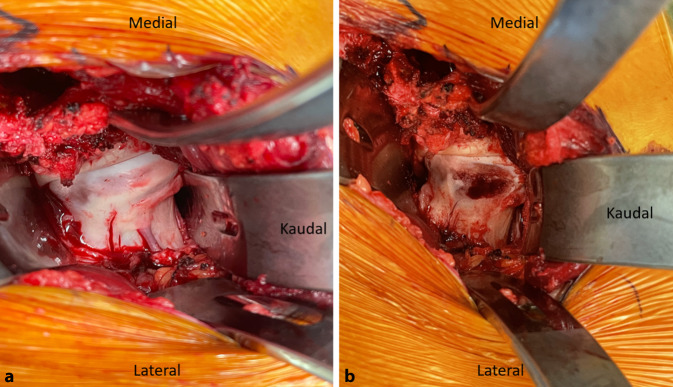

## Besonderheiten

- Ein wichtiger Schritt für eine erfolgreiche Osteotomie ist das Erreichen einer adäquaten Freilegung und Darstellung durch eine ausreichend lange Hautinzision. Wenn die Autoren von einem minimal-invasiven Verfahren sprechen, meinen sie damit die Minimierung des Muskeltraumas und nicht die Minimierung der Länge der Hautinzision. Eine unterdimensionierte Hautinzision kann z. B. aufgrund eines Weichteilkonflikts die retroazetabuläre Osteotomie durch Verdrängen des Osteotoms beeinflussen und so intraartikuläre Frakturen oder eine Fraktur der hinteren Säule begünstigen.
- Die Schambeinosteotomie sollte mit einer kleinen oszillierenden Säge begonnen und mit einem geraden Osteotom abgeschlossen werden, um eine unkontrollierte Spaltung des Schambeins zu vermeiden. Die Schambeinosteotomie sollte ebenfalls nach kaudal und medial gerichtet sein. Eine zu vertikale Ausrichtung der Schambeinosteotomie kann zu einer Lateralisierung des Pfannenfragments führen, wodurch evtl. eine Medialisierung des Schambeins eingeschränkt sein kann.
- Die supraazetabuläre Osteotomie sollte mindestens 3 cm kranial der Hüftgelenkpfanne durchgeführt werden, um genügend Raum für die Insertion der 2 Schanz-Schauben zu schaffen und eine stabile Schraubenfixation zu ermöglichen.
- In Fällen einer weit kaudalen Position der SIAS ist es manchmal notwendig, mit einer zusätzlichen Osteotomie direkt unterhalb der SIAS zu beginnen und diese Osteotomie 1–2 cm nach kranial hinter den Ursprung der SIAS zu verlängern, bevor die horizontale supraazetabuläre Osteotomie durchgeführt werden kann. Dadurch wird gewährleistet, dass genügend supraazetabuläre Substanz und Höhe vorhanden sind, um die 2 Schanz-Schrauben einzubringen.
- Die retroazetabuläre Osteotomie sollte in der Mitte der hinteren Pfannensäule liegen und nicht zu nahe an der Hüftpfanne durchgeführt werden. Der Knochen direkt hinter der Hüftpfanne ist sehr dick und schwierig komplett zu osteotomieren. Wenn die retroazetabuläre Osteotomie nicht vollständig durchgeführt wird und bei der Reorientierung des Azetabulumfragments zu viel Kraft über den Laminaspreizer aufgebracht wird, besteht die Gefahr, eine intraazetabuläre Fraktur durch die Fossa acetabuli zu induzieren.
- Im Falle von Schwierigkeiten bei der Mobilisierung des Pfannenfragments empfehlen die Autoren die Einbringung eines Lambotte-Osteotoms in die retroazetabuläre Osteotomie, um die Kraft während der Reorientierung gleichmäßig auf die gesamte hintere Säule zu verteilen. Dieses Manöver gibt dem Chirurgen eine sehr gute „Rückmeldung“ über die Beweglichkeit des Fragmentes, und bei Bedarf können die unvollständigen Osteotomien überprüft und erneut durchgeführt werden.

## Postoperative Behandlung

**Figure 18:**
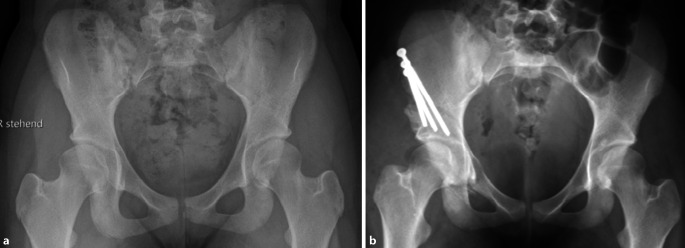

- Als postoperative Ossifikationsprophylaxe werden in Abhängigkeit von ggf. bestehenden Nebenerkrankungen nichtsteroidale Antirheumatika oder ein Coxib für 21 Tage verabreicht. Bis zum Erreichen der Vollbelastung sollte eine medikamentöse Thromboembolieprophylaxe durchgeführt werden.
- Die postoperative Mobilisierung wird im 4‑Punktgang mit 15 kg Belastung der operierten Seite für 4 Wochen durchgeführt. Von der 5. bis zur 7. postoperativen Woche wird im 3‑Punktgang in die schmerzadaptierte Vollbelastung übergegangen.
- Bei ausgeprägten Korrekturen mit großen Osteotomiespalten, wie etwa eine Korrektur einer Hüftdysplasie mit einem LCE unter 0°, wird die postoperative Mobilisierung im 4‑Punktgang mit 15 kg Belastung der operierten Seite für 6 Wochen durchgeführt. Von der 7. bis zur 8. postoperativen Woche wird im 3‑Punktgang in die schmerzadaptierte Vollbelastung übergegangen.
- Um die Stabilität der postoperativen Fixation und die Knochenheilung zu überprüfen, sollte vor der Vollbelastung nach der 4. respektive nach der 6. postoperativen Woche eine a.-p.-Beckenübersichtsröntgenaufnahme angefertigt werden (Abb. 18a). Eine weitere a.-p.-Beckenübersichtsröntgenaufnahme kann bei Bedarf nach dem 3. bis 6. postoperativen Monat durchgeführt werden (Abb. 18b).
- Mit Erreichen der Vollbelastung können leichte sportliche Aktivitäten mit geringer Belastung (z. B. Fahrradergometer) begonnen werden.
- Es bestehen keine Einschränkungen bezüglich des Bewegungsumfanges. Eine physiotherapeutisch angeleitete Mobilisierung kann ab der 1. postoperativen Woche schonend begonnen werden.

## Fehler, Gefahren, Komplikationen

- Gefahr von intraazetabulären Frakturen: Die intraoperative Durchleuchtung ist obligat, um Komplikationen bei der Durchführung einer PAO zu vermeiden. Insbesondere hat sich die Durchleuchtung als zuverlässiges diagnostisches Werkzeug zur Vermeidung von Verletzungen der hinteren Säule und intraartikulärer Osteotomien erwiesen.
- Gefahr von Gefäß- und Nervenverletzungen: Eine adäquate Positionierung des ipsilateralen Beines ist während der Osteotomie von entscheidender Bedeutung, um die Spannung auf die neuralen Strukturen zu minimieren und Verletzungen der Nn. femoralis, ischiadicus und obturator zu vermeiden. Für die Schambeinosteotomie wird das Hüftgelenk in Flexion und Adduktion positioniert. So werden der M. iliopsoas und die neurovaskulären Strukturen medialisiert, was das Risiko für eine Verletzung des N./A./V. femoralis reduziert.
  Verletzung des N. cutaneus femoralis lateralis: Der Nerv wird in jedem Fall in seinem Verlauf und seinen Verzweigungen so weit wie möglich unter der Faszie nach proximal bis zum Leistenband und nach distal präpariert und nach medial mobilisiert. Verletzungen der lateralen und filiformen Äste sind dabei nicht immer zu vermeiden.
- Für den lateralen Anteil der Sitzbeinosteotomie werden Hüftabduktion und -extension empfohlen, um den N. ischiadicus auf die maximale Distanz zum Sitzbein zu bringen. Des Weiteren ist die Osteotomie des Schambeins an der Gegenkortikalis vorsichtig durchzuführen, um ein forciertes Vortreiben und eine Verletzung der Corona mortis, des N. obturatorius und/oder der A. obturatoria zu vermeiden. Außerdem soll das Risiko für diese Komplikationen durch 2 subperiostal um das Schambein eingebrachte, stumpfe Retraktoren vermieden werden. Kommt es hier zu Verletzungen, kann aufgrund ungenügender Visualisierung des Gefäß-Nerven-Bündels eine radiologische Intervention notwendig werden.

## Ergebnisse

In der vorliegenden Arbeit präsentieren wir die Ergebnisse der ersten 39 Patienten (36 Frauen und 3 Männer), die zwischen Januar 2016 und August 2017 vom Seniorautor operativ mittels oben genannter Technik versorgt wurden, zu einem mittleren Follow-up von 3,5 (3 bis 4,5) Jahren. Das durchschnittliche Alter zum Operationszeitpunkt betrug 23 (16 bis 42) Jahre, und der durchschnittliche Body Mass Index (BMI) lag bei 27 (18–36) kg/m^2^. Die durchschnittliche Operationsdauer betrug 88 (57 bis 142) Minuten. Bezüglich der radiologischen Parameter konnten der LCE-Winkel signifikant von präoperativ 16,1° (7–24°) auf postoperativ 30,5° (25–37°) (*p* < 0,0001) und der AC-Winkel signifikant von präoperativ 13,2° (2–25,3°) auf postoperativ 2,8° (−3–13°) (*p* < 0,0001) korrigiert werden.

In 20 von 39 Fällen (51,3 %) wurde eine Hypästhesie durch eine Schädigung des NCFL am lateralen Oberschenkel registriert. Neben der Verletzung des NCFL wurden keine weiteren Nervenverletzungen und keine Gefäßkomplikationen beobachtet. Bei keinem der Patienten war die Transfusion von Erythrozytenkonzentraten notwendig. Es zeigten sich keine ungewünschten intra-/postoperativen Frakturen, und es kam zu keinem postoperativen Korrekturverlust. Es kam in 3 Fällen (7,7 %) zu einer Über‑/Unterkorrektur der dreidimensionalen Korrektur. In einem Fall wurde das Azetabulum überkorrigiert mit einem LCE-Winkel von 37° und einem AC-Winkel von −3°. Bei 2 Patienten wurden postoperative azetabuläre Retroversionen registriert. Bei einem dieser Patienten wurde die präoperativ vorliegende Retroversion nicht ausreichend korrigiert. Bei dem zweiten Patienten zeigte sich postoperativ bis zum letzten Follow-up eine vermehrte postoperative Beckeninklination auf der a.-p.-Beckenübersichtsaufnahme. Dadurch entstand eine funktionelle Überkorrektur der anterioren Überdachung.
